# Response Enhancement of Pt–AlGaN/GaN HEMT Gas Sensors by Thin AlGaN Barrier with the Source-Connected Gate Configuration at High Temperature

**DOI:** 10.3390/mi12050537

**Published:** 2021-05-10

**Authors:** Tuan-Anh Vuong, Ho-Young Cha, Hyungtak Kim

**Affiliations:** School of Electronic and Electrical Engineering, Hongik University, Seoul 04066, Korea; tuananhvuong2017@gmail.com (T.-A.V.); hcha@hongik.ac.kr (H.-Y.C.)

**Keywords:** gallium nitride, hydrogen sensing, two-terminal HEMT, high temperature, harsh environment, gate-recess, normally-on

## Abstract

AlGaN/GaN HEMT hydrogen gas sensors were optimized by AlGaN barrier thickness in the gate-source connected configuration demonstrated high response and robust stability up to 500 °C. First, we found that the hydrogen sensing performance of a conventional normally-on HEMT-based sensor was enhanced when zero voltage was applied on the gate in comparison with a floating-gate condition due to a reduced level of the base current. In the next step, to take advantage of the response increase by V_GS_ = 0 V, a new type of sensor with a source-connected gate (SCG) was fabricated to utilize the normally-on operation of the GaN HEMT sensor as a two-terminal device. AlGaN barrier thickness was thinned by the dry-etching process to gain higher transconductance at a zero-gate bias with the reduction of the distance from the 2DEG channel to the AlGaN surface, thereby significantly improve the hydrogen response. The SCG GaN sensor with an ultra-thin AlGaN barrier (9 nm) exhibited responses of 85% and 20% at 200 and 500 °C, respectively, onto 4%-hydrogen gas, which demonstrates a promising ability for harsh environment applications.

## 1. Introduction

Hydrogen gas is considered as an energy carrier in the modern world, and possesses outstanding properties and features that make it become a very promising source of energy for automotive or fuel cell applications, although it is not naturally available as a ready-to-use substance [1,2,3]. However, when using hydrogen gas at high temperatures, there emerge two main concerns, including high-temperature hydrogen attack (at least 200 °C), and auto-ignition (at 500 °C). According to that reason, detection of hydrogen leaks is very important, especially at high temperatures. Gallium nitride possesses a wide bandgap of 3.2 eV and a high critical electric field of 3 MV/cm, thereby leading the device operation with a very low leakage current in high temperatures and extreme environments. The AlGaN/GaN heterojunction also induces two-dimensional electron gas (2DEG) with high electron mobility, which enables a prompt response. Therefore, GaN heterojunction-based sensors can be a strong candidate for a hydrogen sensor platform that requires a high temperature operation for an active catalytic reaction in hydrogen, which is well above the temperature limitation of silicon devices. Pt/AlGaN/GaN-based sensors have been widely investigated because they demonstrated the potential to respond towards the hydrogen presence [4,5,6,7]. When the H_2_ molecules are adsorbed onto the Pt surface, they are decomposed into hydrogen atoms, penetrate through the Pt layer, and form an interfacial dipole layer at the metal–semiconductor interface. The dipole layer reduces the Schottky barrier height and induces the negative shift of pinch-off voltage, thus increasing the drain current [8,9,10]. Consejo et al. reported experimental results indicating that a change of channel conductivity by hydrogen incorporation is mainly caused by carrier density change, and the change of carrier mobility has a negligible effect [11]. In many literatures, hydrogen gas sensors with AlGaN/GaN heterostructures employed Schottky diodes or floating-gate HEMTs [12,13,14,15,16,17,18,19]. Although a HEMT-type device with three terminals, including the source, drain, and gate, can have an advantage of modulating the response by tuning gate bias [20], it requires an additional interconnection and eventually increases the complexity of integration between sensor arrays and readout circuitry. Two-terminal GaN HEMT sensors with a floating Pt-gate also demonstrated H_2_ sensing performance [21,22], but they cannot take advantage of gate modulation to enhance the response. GaN HEMTs are normally-on devices in which the channel current conducts at V_GS_ = 0 V due to the 2DEG channel being induced at the AlGaN/GaN interface. Zero-gate potential can be easily assigned by connecting the gate and source electrodes as a common electrode, and this configuration is attractive when the sensor performance is optimized at V_GS_ = 0 V. In this work, the enhanced response was achieved when the transconductance (g_m_ $=\frac{\partial I_{DS}}{\partial V_{GS}}$) was increased at V_GS_ = 0 V by thinning the AlGaN barrier, because 2DEG is very sensitive to the surface potential.

## 2. Experimental

**Figure 1:**
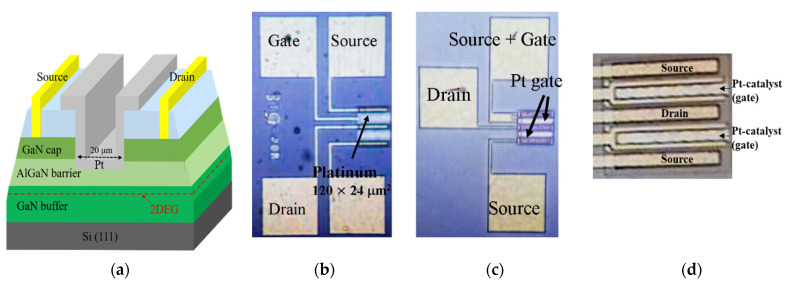
(**a**) The schematic cross-sectional 3D diagram of the recessed AlGaN/GaN HEMT-based sensors on a Si substrate. In standard (no-recess) sensors, the layers under the gate region are in situ SiN_x_, GaN cap, and AlGaN, respectively. W_G_/L_G_ = 120 μm/24 μm. (**b**) Microscopic image of a 3-terminal standard HEMT sensor (upper) and a floating gate sensor (lower). (**c**) Microscopic image of a 2-terminal SCG sensor (upper). (**d**) Magnified image of active area showing Pt-catalyst.

Pt-gate AlGaN/GaN HEMT gas sensors were fabricated on commercially-available AlGaN/GaN-on-Si substrate at the Inter-University Semiconductor Research Center, Seoul, Korea. The epitaxial structure consists of a 4-μm GaN buffer layer on a (111)-oriented silicon substrate, a 23-nm AlGaN barrier layer, a 4-nm GaN cap layer, and a 10-nm in situ SiNx passivation layer on the top. The source and drain contacts with Ti/Al/Ni/Au (200/1200/250/500 Å) were formed by e-beam evaporation followed by rapid thermal annealing (RTA) at 830 °C for 30 s in N_2_ ambient. Mesa isolation with 400-nm depth was formed by inductively-coupled-plasma reactive-ion-etching (ICP-RIE) with BCl_3_/Cl_2_ to define the active region. A 30 nm-thick Pt-sensing area was formed by e-beam evaporation and lift-off process. For sensor devices with thinner AlGaN barrier thicknesses, the AlGaN layer under the gate region was partially recessed using ICP-RIE with BCl_3_/Cl_2_ prior to Pt-gate deposition. The recess depth of AlGaN was confirmed by atomic force microscopy (AFM) measurement. Finally, the probing pads were formed with Ni/Au (=20/250 nm) evaporation. Figure 1a shows the schematic 3D diagram of the fabricated sensor with the partially-recessed AlGaN barrier. Figure 1b presents a top-view image of a 3-terminal sensor (upper) and a floating-gate sensor (lower part). Figure 1c shows the image of a 2-terminal source-connected gate (SCG) sensor (upper part). Hydrogen sensing characteristics were measured in the gas chamber probe station with gas flows of 4%-H_2_ in argon background gas at high temperatures beyond 200 °C using an Agilent 4155A parameter analyzer. The flow rate was set to 1000 sccm. The SCG sensors were designed and fabricated after the effect of zero-gate bias was found from the 3-terminal sensors.

Hydrogen sensing characteristics were measured in the gas chamber probe station with exposure to gas flows of 4%-H_2_ in argon background gas by an MFC gas controller at elevated temperatures using an Agilent 4155A parameter analyzer.

## 3. Results and Discussion

**Figure 2:**
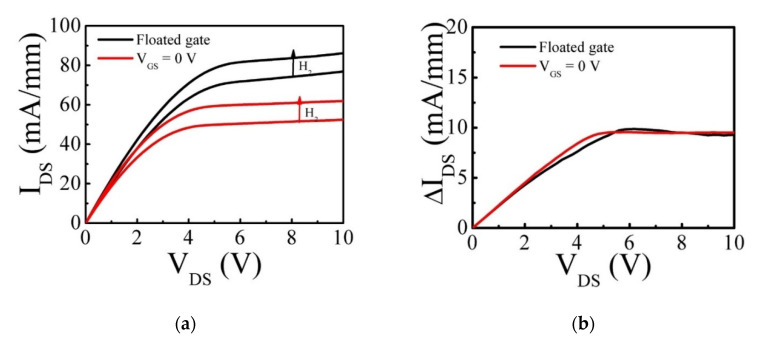
(**a**) Output characteristics of a 3-terminal standard Pt/AlGaN/GaN HEMT-based sensor (no recess) in the air and in H_2_ at 200 °C with a floating gate and V_GS_ = 0. (**b**) ΔI_DS_ = I_H_2__ − I_air_ for each case.

**Figure 3:**
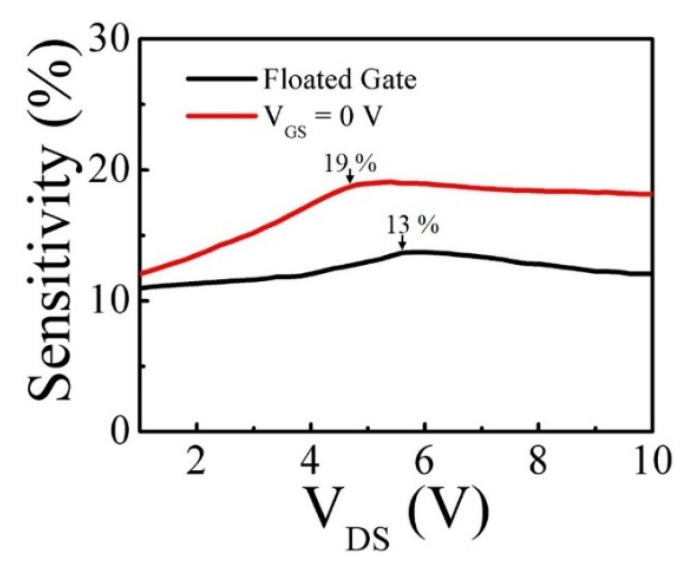
The comparison of responses between a floating gate and V_GS_ = 0 V of a standard Pt/AlGaN/GaN HEMT-based sensor.

When the sensors were exposed to H_2_ gas flow, an interfacial dipole layer was formed at the Pt/AlGaN interface and reduced the Schottky barrier height, resulting in an increase of the channel current (I_DS_), as shown in Figure 2a. The output characteristics of a standard (no-recess) HEMT sensor showed that I_DS_ was increased by H_2_ exposure. The response in percentage was defined as (I_H2_ − I_air_)/I_air_, where I_H2_ and I_air_ are the I_DS_ measured with and without H_2_ exposure, respectively. Interestingly, the maximum response was improved from 13 to 19% at V_GS_ = 0 V in comparison to the gate floated, as shown in Figure 3. This response enhancement can be explained by Figure 2b, indicating that the amount of the current change (ΔI_DS_) by H_2_ detection was not different, although the drain current was decreased by a zero-gate voltage.

**Figure 4:**
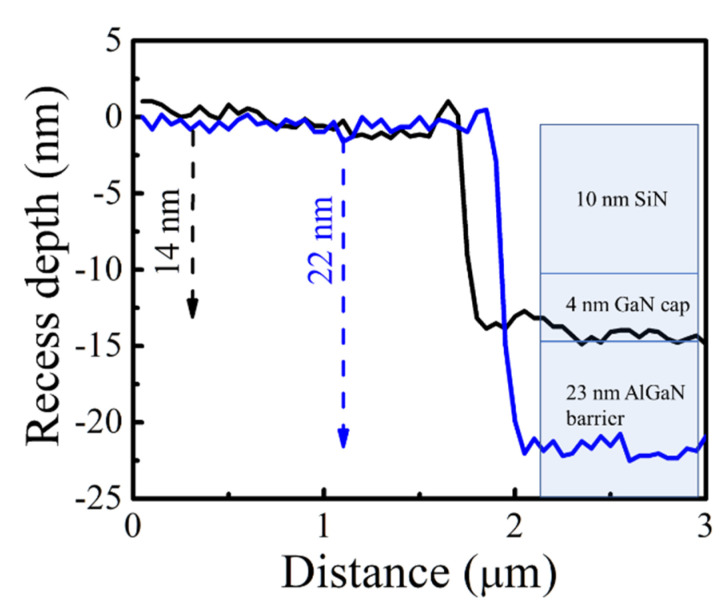
The etch depth of the gate region recess measure by AFM. The etch depth of 22 nm corresponds to the remaining AlGaN thickness of 15 nm.

**Figure 5:**
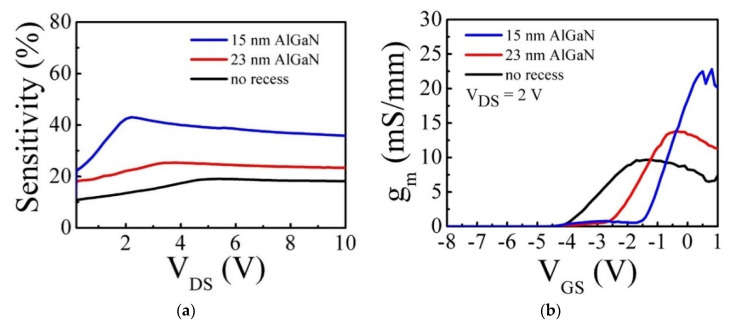
The dependence of (**a**) response with V_GS_ = 0 V and (**b**) transconductance on AlGaN barrier thickness. Gas condition: 4%-H_2_ in argon background gas, 200 °C.

**Figure 6:**
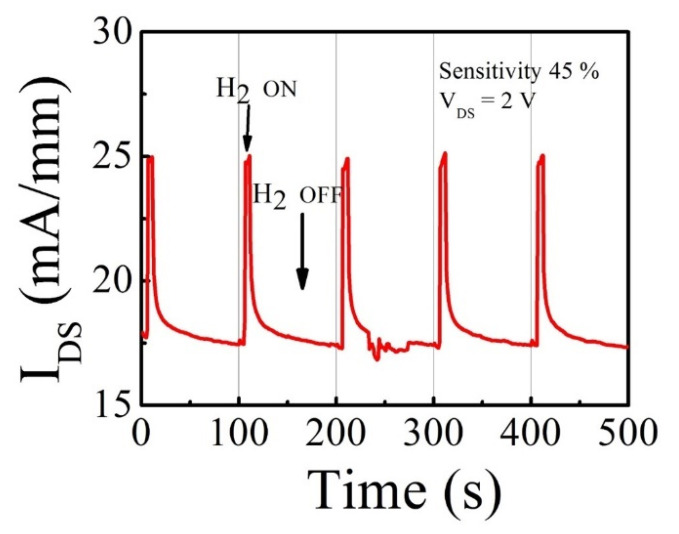
Transient-response characteristics to hydrogen of a 3-terminal standard sensor with 15 nm-AlGaN measured with V_GS_ = 0 V, V_DS_ = 2 V at 200 °C—one cycle included 4% hydrogen in argon injection for 5 s followed by a recovery period.

The g_m_ is a key parameter of HEMT devices, demonstrating how well gate potential can control the channel conductivity. Therefore, the HEMT-type sensors with high g_m_ are expected to become highly sensitive to the change of the surface potential by H_2_ detection. We carried out recess etching using ICP-RIE in the gate area to reduce the AlGaN barrier thickness to improve the response further. The sensors with 23 and 15 nm AlGaN barriers were fabricated by adjusting the etching time on the same wafer. The etch depth was measured by atomic force microscopy (AFM), as seen in Figure 4. The etch depth of 22 nm resulted in a 15 nm-AlGaN barrier. As shown in Figure 5a, the hydrogen response at V_GS_ = 0 V was increased as the barrier thickness was reduced. Table 1 contains the maximum response values measured from each sensor, and the highest response of 42% was observed from the sensor with a 15 nm-AlGaN barrier. Figure 5b is the g_m_ of the sensors with three different AlGaN thicknesses. As presented in Table 1, maximum g_m_ obviously increased from 19 mS/mm of non-recessed sensors to 42 mS/mm of 15 nm-AlGaN sensors, suggesting that a strong correlation exists between the response and the g_m_, which indicates the controllability of the gate potential, i.e., surface potential over the electron channel. Therefore, the sensor with high g_m_ is very sensitive to the change of the surface potential induced by gas detection. Figure 6 shows the transient-response characteristic of the sensor with 15 nm AlGaN barrier thickness. One cycle consists of 4% hydrogen in argon injection for 5 s followed by a recovery period. The result suggests that the fabricated sensors can operate stably when they are exposed to cyclic hydrogen gas flows at 200 °C.

**Figure 7:**
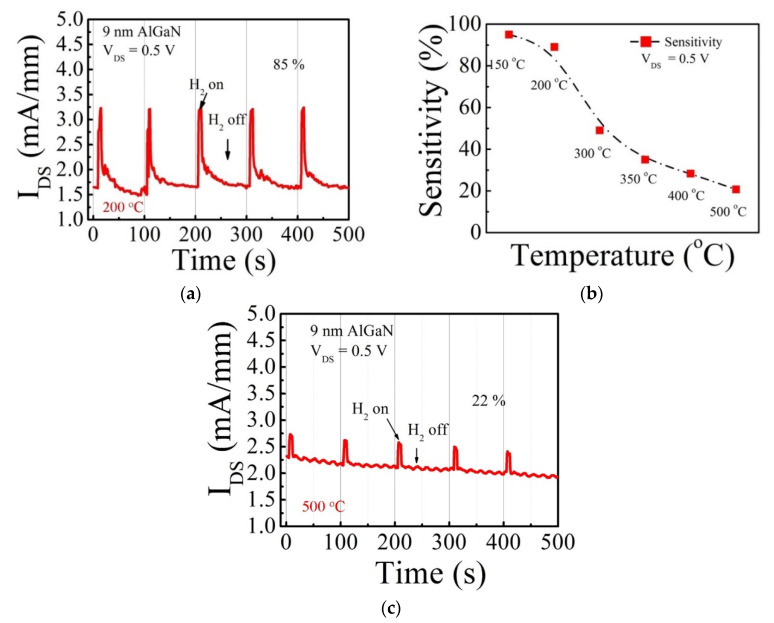
(**a**) Transient-response characteristics at 200 °C of a 2-terminal SCG sensor with a 9 nm-AlGaN barrier. (**b**) The dependence of response on the temperature increase. (**c**) Transient-response characteristics at 500 °C. One cycle with 4% hydrogen in argon injection for 5 s followed by a recovery period.

When the unit sensor devices are integrated with a readout circuit or built in the arrays, the sensors with a smaller number of the terminals are preferred in terms of a complexity. The FET-type sensors can take advantage of the gate modulation of the output current, and the optimized gate bias for the sensitivity can be applied. This requires additional voltage generation in the operation circuit. If the HEMT-type sensors can be optimized at zero gate bias by changing AlGaN barrier thickness, they can work in the source-connected gate configuration with an enhanced sensitivity and no additional voltage generation. To take an advantage of response enhancement by V_GS_ = 0 V, and also to simplify the circuit design, a SCG sensor was fabricated by connecting the source and gate electrodes together, thereby making a two-terminal HEMT (Figure 1c). SCG sensors can still conduct the channel current at V_GS_ = 0 V because GaN HEMT is a normally-on (or depletion-mode) FET device. The gate recess etching was carried out, and the 9 nm-AlGaN thickness was measured in this process run. Figure 7 shows the transient-response characteristics measured from the two-terminal SCG sensor with a 9-nm AlGaN barrier. In comparison with the result in Figure 6, the response was even more improved up to 85% by reducing the AlGaN thickness from 15 to 9 nm. The hydrogen transient-response characteristics were also measured from 150 to 500 °C because the GaN-based sensor is a strong candidate for harsh environment application. As shown in Figure 7b, the response was highest at 150 °C, then decreased as temperature rose to 500 °C. However, the sensor still demonstrated a sound stability at 500 °C with a 22%-response, as shown in Figure 7c. This result suggests that two-terminal GaN SCG HEMT sensors have a strong capability of hydrogen sensing at very high temperatures. In Table 2, GaN-based hydrogen sensor characteristics are summarized for comparison. Our SCG sensors demonstrated excellent sensor response characteristics as well as high temperature capability.

## 4. Conclusions

Pt–AlGaN/GaN HEMT sensors for hydrogen detection fabricated on AlGaN/GaN-on-Si demonstrated strong ability in the detection of hydrogen gas at high temperatures. The improvement of response by applying 0 V at the Pt-gate electrode was observed because zero gate bias reduced the baseline drain current without changing the difference of the channel current responding to hydrogen. The advantage of applying V_GS_ = 0 V was taken by connecting source and gate electrodes to create a two-terminal SCG sensor with a normally-on GaN HEMT. Further enhancement of the response was observed from the sensors with a thinner AlGaN barrier due to the increased transconductance. For SCG sensors, the highest hydrogen response of 85% was obtained from a 9-nm AlGaN barrier SCG sensor. This sensor also demonstrated stable operation at 500 °C.

## Figures and Tables

**Table 1:** Maximum response and transconductance dependence on AlGaN barrier thickness.

AlGaN Thickness	No Recess	23 nm	15 nm
Response (%)	19	25	42
g_m_ (mS/mm)	10	13.3	22.5

**Table 2:** Summary of the GaN-based sensor characteristics with previous reports.

Sensor Platform	Temp.	Hydrogen Concentration	Response Time	Recovery Time	Sensor Response	Ref.
Diode (GaN)	Room temp.	1%	15 s	19 s	1 × 10^5^%	[15]
Diode (GaN)	200 °C	4%	-	-	7 × 10^8^%	[16]
Diode (GaN)	300 °C	0.081%	25.1 s	34.1 s	0.11%	[17]
Diode (AlGaN/GaN)	Room temp.	0.05%	-	-	2.4%	[18]
Diode (AlGaN/GaN)	Room temp.	4%	-	-	3700%	[19]
MOS Diode (AlGaN/GaN)	Room temp.	10%	~30 s	-	-	[7]
HEMT (Pd–AlGaN/GaN)	200 °C	4%	3 s	-	72%	[21]
HEMT (Pd–AlGaN/GaN)	200 °C	4%	<0.4 s	12.4 s	80%	[23]
HEMT (Pt–AlGaN/GaN)	200 °C 500 °C	4%	3 s <1 s	20 s 4 s	85% 22%	This work
